# The Cross-Sectional Relations of COVID-19 Fear and Stress to Psychological Distress among Frontline Healthcare Workers in Selangor, Malaysia

**DOI:** 10.3390/ijerph181910182

**Published:** 2021-09-28

**Authors:** Natasha Subhas, Nicholas Tze-Ping Pang, Wei-Cheng Chua, Assis Kamu, Chong-Mun Ho, Isabel Shamini David, William Wei-Liang Goh, Yogaraja Indran Gunasegaran, Kit-Aun Tan

**Affiliations:** 1 Department of Psychiatry and Mental Health, Hospital Tengku Ampuan Rahimah, Jalan Langat, Klang 41200, Malaysia; yogarajaindran03@gmail.com; 2 Faculty of Medicine and Health Sciences, Universiti Malaysia Sabah, Kota Kinabalu 88400, Malaysia; weicheng921@gmail.com (W.-C.C.); assis@ums.edu.my (A.K.); cmho@ums.edu.my (C.-M.H.); 3 Department of Psychiatry and Mental Health, Hospital Banting, Banting 42700, Malaysia; isabelshamini@gmail.com; 4 Department of Psychiatry and Mental Health, Hospital Segamat, KM6, Segamat 85000, Malaysia; william89goh@gmail.com; 5 Department of Psychiatry, Faculty of Medicine and Health Sciences, Universiti Putra Malaysia, Serdang 43400, Malaysia; tanka@upm.edu.my

**Keywords:** COVID-19 fear, COVID-19 stress, anxiety, depression, psychological distress, frontline healthcare workers

## Abstract

Previous pandemics have demonstrated short and long-term impacts on healthcare workers’ mental health, causing knock-on effects on patient care and professional functioning. Indeed, the present COVID-19 pandemic has created unprecedented disruption in social interactions and working conditions. Malaysia has been under the Recovery Movement Control Order since June 2020; however, with the upsurge of cases, healthcare workers face pressure not only from working in resource-deprived settings but also from the increasing patient load. The primary objective of the present study was to examine the cross-sectional relationship of COVID-19 fear and stress to psychological distress (operationalized as anxiety and depression) in healthcare workers. The present sample included 286 frontline healthcare workers from three hospitals in Selangor, Malaysia. Self-administered questionnaires containing sociodemographic and occupational items, the Malay versions of the Coronavirus Stress Measure scale, the Fear of Coronavirus-19 scale, the Generalized Anxiety Disorder-7, and the Patient Health Questionnaire-9 were distributed via online platforms. Hierarchical multiple regression findings suggest that age, shift work, and COVID-19 stress consistently predicted anxiety and depression among frontline healthcare workers after adjusting for sociodemographic and occupational variables. The present findings suggest that frontline healthcare workers are not only inoculated against COVID-19 itself but also against the psychological sequelae of the pandemic.

## 1. Introduction

The severe acute respiratory syndrome coronavirus 2 (SARS-CoV-2) and coronavirus disease (COVID-19) pandemic has created unprecedented disruption in social interactions and working conditions, both cross-sectionally and longitudinally [1,2,3,4]. The sudden outbreak of this highly contagious disease is unpredictable and is associated with high mortality and morbidity [5], causing a tremendous toll on the population, including psychological distress (operationalized as anxiety and depression) [6,7]. Unique challenges to healthcare workers were presented at the beginning as treatment courses were still under investigation and vaccines were not yet produced [5,8]. Hence, there was great fear of COVID-19 not just for patients but also for healthcare workers’ health and that of their close family members and friends [5]. The previous H1N1 influenza pandemic, Middle East respiratory syndrome, severe acute respiratory syndrome, H5N1 avian flu, and West Nile fever outbreaks demonstrated short and long-term impacts on healthcare workers’ mental health, which could have knock-on effects on patient care and professional functioning [5].

Malaysia first documented the first case of COVID-19 on 25 January 2020, and it was traced back to Chinese nationals. A nationwide Movement Control Order was imposed on 18 March 2020 due to the sudden surge of cases and to control the spread of COVID-19 cases nationwide [8]. Since then, the Malaysian government has placed the country under the Conditional Movement Control Order from 13 May to 9 June, followed by the Recovery Movement Control Order from 10 June to 31 December 2020 [9]. However, as of early January 2021, cases are beginning to increase once more. Healthcare workers will again be facing the pressure of working in resource-deprived settings with an ever-growing patient load, not only in Malaysia but worldwide [10,11]. Systematic reviews have suggested that risk factors contributing to poor mental health outcomes for healthcare workers include their level of exposure to the disease, being quarantined, and personal health fears [12]. Sociodemographic factors—younger age, being female, and having a low monthly household income—were found to be correlated with psychological distress [5,13,14,15]. Occupational factors such as employment status, shift work, and years of employment could also contribute to anxiety and depression in healthcare workers [16,17,18]. As for employment status, healthcare workers who had statutory fixed-term employment (permanent staff) were found to be more likely to suffer from anxiety and depression during the pandemic [19]. With respect to shift work, healthcare workers gave feedback that they had poor concentration due to prolonged work hours, and this invariably gave them anxiety and depression [20]. Psychological distress was associated with those having more years of employment [17,21].

Two new psychopathological constructs that have surfaced from the COVID-19 pandemic are “fear of COVID-19” and “stress of COVID-19”. Fear of COVID-19 was constructed to address a specific characteristic nature of the infectious disease, namely fear, which can be associated with the pandemic transmission rate and medium (rapidly and invisibly), morbidity, and mortality (henceforth referred to as “COVID-19 fear”) [22,23]. Among other scales, the Fear of COVID-19 Scale has been adapted into multiple languages and validated with replicable and acceptable psychometric properties [24,25,26,27]. From an evolutionary perspective, psychological distress occurs in individuals who have a fear of harm to self (e.g., fear of dying or getting in contact with individuals who are possibly infected) [22,23]. There were local studies documenting the cross-sectional relationship of COVID-19 fear and psychological distress (i.e., anxiety and depression) in Malaysian populations; however, only bivariate findings were available with COVID-19 fear operationalized as an independent variable and psychological distress as a dependent variable [13,14]. This suggests an urgent need to examine such relationships with multivariate analyses.

Stress associated with COVID-19 has been defined as a specific, stress-related response to the constellation of factors surrounding the pandemic (henceforth referred to as “COVID-19 stress”) [28]. The five-item Coronavirus Stress Measure (CSM) was adapted from the Perceived Stress Scale and was specifically designed to provide a shorter stress scale that could rapidly measure stress related to COVID-19 compared to the 14-item Perceived Stress Scale and the 36-item COVID Stress Scale [28,29]. There were previous findings documenting the relationships among COVID-19 stress, psychological distress (i.e., anxiety and depression), and psychological inflexibility; however, such findings were only available in western populations (e.g., Canada and the USA) [30].

Locally, to date, there are limited Malaysian data on the relationships between the two new COVID-19 psychopathological constructs and psychological distress in healthcare workers. The prevalence of risk of depression was found to be 67.1% in anesthetists from a Malaysian COVID-19 referral hospital [31]. In other studies, the prevalence rates ranged from 11.1% to 31.6% for anxiety and from 9.9% to 21.8% for depression [32,33]. Only large-scale data involving local, non-frontline university populations are currently available [13,14]. Healthcare workers employed at non-frontline university hospitals are reported to be less susceptible to burnout and overloading compared to those working at national hospitals. There exists an urgent need to enrich our scientific knowledge base by recruiting samples from national hospitals that are expected to have high patient volume, morbidity, and mortality [34]. Therefore, the primary objective of the present study was to examine the relationships of COVID-19 fear and stress to anxiety and depression in a sample of frontline healthcare workers from national hospitals. We hypothesized that COVID-19 fear and stress would account for a significant amount of variance in anxiety and depression over and above that accounted for by sociodemographic (age, gender, and monthly household income) and occupational (employment status, shift work, and years of employment) factors.

## 2. Materials and Methods

The present study was cross-sectional by design. We employed a universal sampling technique to obtain the present sample. The non-response rate was 48%. The present sample included 286 healthcare workers (80% female and 20% male) from three hospitals in Selangor, a state in Malaysia. They were only included in the present study if they were classified as frontline healthcare workers (i.e., they had direct or indirect involvement with taking care of COVID-19 patients), could read and understand the Malay language, and were ≥18 years of age. Administration and non-clinical staff were excluded from the present study.

### 2.1. Participants

The present sample was predominantly Malay (71.7%) and Muslim (75.9%). These demographics are representative of national statistics on the total population in Malaysia [35]. The mean age of the present sample was 33.02 years (*SD* = 6.79), ranging from 23 to 57 years. We also found that 97.9% of the participants had completed a diploma or bachelor’s degree, 71.7% were married, 52.1% lived less than 10 km away from their hospitals, 54% had less than MYR 5,000 for household income per month, 72% did not have a history of medical problems, and 94.1% did not have a history of psychiatric problems. As far as occupational information is concerned, 84.3% of the participants were permanent staff, 51.7% were working in shifts, and 73.5% had less than 10 years of employment history.

### 2.2. Measures

Participants completed a Google form consisting of sociodemographic and occupational items, the Malay versions of the Coronavirus Stress Measure Scale, the Fear of Coronavirus-19 Scale, the Generalized Anxiety Disorder-7, and the Patient Health Questionnaire-9. This form was distributed from 23 November 2020 to 31 December 2020 via social media platforms (e.g., WhatsApp, official hospital Facebook accounts, and Messenger). Participation was strictly voluntary and took 5 to 10 min to complete. Participants were asked to ignore the Google form invitation if they did not want to participate in the study. Informed consent was obtained prior to the commencement of data collection.

#### 2.2.1. Sociodemographic and Occupational Items

Sociodemographic items included age (continuous), gender (1 = Female, 0 = Male), ethnicity (5 = Malay, 4 = Chinese, 3 = Indian, 2 = Bumiputera Sabah, 1 = Bumiputera Sarawak, 0 = Others), religion (4 = Islam, 3 = Buddhism, 2 = Hinduism, 1 = Christianity, 0 = Others), level of education (2 = at least a bachelor’s degree, 1 = diploma, 0 = secondary school), marital status (2 = single, 1 = married, 0 = divorced), distance from place of residence to work (3 = more than 30 km, 2 = 20 to 30 km, 1 = 10 to 20 km, 0 = less than 10 km), household income per month (continuous), and history of a medical (1 = yes, 0 = no) or psychiatric problem (1 = yes, 0 = no). Occupational items obtained information on contact with COVID-19 patients (1 = direct, 0 = indirect), employment status (1 = permanent staff, 0 = contract staff), shift work (1 = yes, 0 = no), and years of employment (continuous).

#### 2.2.2. The Malay Version of the Fear of Coronavirus-19 Scale

The seven-item Malay version of the Fear of Coronavirus-19 (FC) scale is a self-measure of fear resulting from COVID-19 [19]. Participants rate items on a five-point Likert scale ranging from 1 (strongly disagree) to 5 (strongly agree). Higher scores indicate greater COVID-19 fear. Cronbach’s alpha was 0.89 for the Malay version of the FC in the present study.

#### 2.2.3. The Malay Version of the Coronavirus Stress Measure Scale

The five-item Malay version of the Coronavirus Stress Measure (CSM) scale is designed to measure stress in response to COVID-19 [36]. Participants rate items on a five-point Likert scale ranging between 0 (never) and 4 (very often). Higher scores indicated greater COVID-19 stress. Cronbach’s alpha was 0.89 for the Malay version of the CSM in the present study.

#### 2.2.4. The Malay Version of the Generalized Anxiety Disorder-7

The Malay version of the Generalized Anxiety Disorder-7 (GAD-7) is a seven-item scale designed to measure anxiety [37]. Participants rate items on a four-point Likert scale ranging from 0 (not at all) to 3 (nearly every day). Higher scores indicate greater anxiety. Cronbach’s alpha was 0.94 for the Malay version of the GAD-7 in the present study.

#### 2.2.5. The Malay Version of the Patient Health Questionnaire-9

The Malay version of the Patient Health Questionnaire-9 (PHQ-9) is a nine-item scale designed to measure depression [38]. Participants rate items on a four-point Likert scale ranging from 0 (not at all) to 3 (nearly every day). Higher scores indicate greater depression. Cronbach’s alpha was 0.88 for the Malay version of the PHQ-9 in the present study.

### 2.3. Data Analysis Strategy

We used IBM SPSS version 27 (IBM Corporation, New York, NY, USA) to perform data analyses. Descriptive statistics were used to describe study variables. We inspected skewness (±3)  and kurtosis (±10)  indices for the normality assumption [39]. If this assumption was not met, the data were transformed to achieve normality (i.e., log10 transformation for positively skewed data and square root transformation for negatively skewed data) [40].

We performed a series of one-way analyses of variance (ANOVA) to examine the differences in anxiety and depression according to sociodemographic (ethnicity, religion, level of education, marital status, distance from place of residence to work, history of a medical problem, and history of a psychiatric problem) and occupational (contact with COVID-19 patients) items. Pearson’s *r* correlation coefficients were performed to examine the relationship among study variables at a bivariate level. Possible multicollinearity between each pair of independent variables was considered if the *r* correlation coefficients were above 0.70 [41].

Separate hierarchical multiple regression analyses were performed with anxiety and depression as dependent variables. In Step 1, sociodemographic (age, gender, and monthly household income) and occupational factors (employment status, shift work, and years of employment) were entered. In Step 2, COVID-19 fear and COVID-19 stress were entered. We inspected variance inflation factors (VIF; >10) and tolerance (<0.01) values for multicollinearity assumptions [42].

## 3. Results

### 3.1. Descriptive and Bivariate Findings

Table 1 presents the descriptive and bivariate statistics for the variables in the analysis. There was no violation of the normality assumption as all skewness and kurtosis indices were within the acceptable range, except for monthly household income and years of employment [38]. These two variables were positively skewed and were log10-transformed for all subsequent analyses.

One-way ANOVAs were performed to examine the differences in anxiety and depression depending on sociodemographic and occupational items. Significant differences in anxiety were identified between religion, *F* (19, 266) = 1.85, *p* = 0.02; level of education, *F* (19, 266) = 1.81, *p* = 0.02; distance from place of residence to work, *F* (19, 266) = 1.94, *p* = 0.01; and history of a psychiatric problem, *F* (19, 266) = 7.48, *p* < 0.001. Depression also significantly differed between religion, *F* (24, 261) = 2.14, *p* = 0.002, and history of a psychiatric problem, *F* (24, 261) = 1.64, *p* = 0.03.

All significant correlations among COVID-19 fear, COVID-19 stress, anxiety, and depression were found to be in the expected directions. Years of employment was excluded from the hierarchical regression analyses due to a high correlation with age (*r* = 0.77).

### 3.2. Multivariate Findings

Table 2 and Table 3 present the results of hierarchical regression analyses predicting anxiety and depression, respectively. Across all regressions, VIF and tolerance values were found to be within the acceptable range. No violation of multicollinearity was detected [42].

#### 3.2.1. Predicting Anxiety

In Step 1, only age and shift work contributed significantly to the prediction of anxiety, *F* (5, 279) = 3.84, *p* < 0.01. In Step 2, age, shift work, and COVID-19 stress significantly predicted anxiety, *F* (7, 277) = 24.72, *p* < 0.001, accounting for 38% of the variance in anxiety. Younger age, shift work, and having greater COVID-19 stress contributed to anxiety.

#### 3.2.2. Predicting Depression

In Step 1, only age and shift work contributed significantly to the prediction of depression, *F* (5, 279) = 5.07, *p* < 0.001. In Step 2, age, shift work, and COVID-19 stress significantly predicted depression, *F* (7, 277) = 21.08, *p* < 0.001, accounting for 35% of the variance in depression. Younger age, shift work, and having greater COVID-19 stress contributed to depression.

## 4. Discussion

Based on existing literature, the present study examined the relative contribution of sociodemographic and occupational factors and COVID-19 fear and stress on psychological distress, namely in the form of anxiety and depression, in a sample of frontline healthcare workers from Malaysian national hospitals. Across psychological distress variables, age, shift work, and COVID-19 stress emerged as consistent predictors. As age was unmodifiable, our hypothetical figure was that future mental health research could target shift work and COVID-19 stress in an attempt to allay anxiety and depression in frontline healthcare workers. We offer some suggestions below.

First, the findings for shift work go against overwhelming evidence linking it to negative psychological sequelae [43,44]. COVID-19 stress is new and interminable [45,46], with unrelenting patient care, especially evident in a non-shift environment where on-call and pro bono overtime work are the norm. There may exist other mediators that could alter the relationship between shift work and psychological distress, which awaits future investigation. Nonetheless, shift work, despite the inevitable disruptions to sleep and wake cycles, may actually be preferable for some as it allows exhausted frontline health workers to rest and recharge in shifts and share the burden of patient care over a corresponding 24-h period, reducing the probability of vicarious trauma [47,48,49].

Second, our hierarchical multiple regression findings show that COVID-19 fear did not significantly predict anxiety and depression. Psychological distress is evident in individuals who a have fear of harm to self (from an evolutionary perspective) in the face of the COVID-19 pandemic (i.e., COVID-19 fear) [50,51]. Previous studies in a non-healthcare worker population suggested that COVID-19 fear was related to anxiety and depression at a bivariate level [13]. Nonetheless, this is the first study to extend previous studies [14] examining the relationship of COVID-19 fear to psychological distress at a multivariate level. Future research should define the role of COVID-19 fear both theoretically and practically in order to enrich our understanding of how the pandemic could affect not only professional functioning but also the psychological well-being of frontline healthcare workers.

Third, our findings add to the COVID-19 literature delineating the developmental process from COVID-19 stress to anxiety and depression. COVID-19 stress was found to be prevalent in frontline healthcare workers as they were aware of the health risks [52], coupled with high levels of exposure to death, disability, and trauma, as observed at COVID-19 intensive care units and medical wards, which in turn linked to psychological distress [53]. Since the outbreak of COVID-19, the psychological support systems in most mental health institutions had to be modified in order to reach distressed healthcare workers and abide by the local standard operating procedures [54,55]. With the advancement of social media, psychological support services targeted specific interventions and consultations for distressed healthcare workers, using online platforms, telehealth, or mobile apps, all of which could then be delivered worldwide [54]. Malaysia is no exception. For example, the Malaysian mental health and psychosocial services provide psychological assessments, interventions, and first aid to at-risk groups via telepsychiatry services using audio or video calling [55]. From a cognitive perspective, it is plausible that the COVID-19 pandemic would unduly affect cognitive patterns by increasing the inherent uncertainty present in wards, which in turn would have knock-on effects on psychological distress as an intrapsychic response to uncertainty. Indeed, COVID-19 stress represents a new and emerging cognitive factor that has the potential to contribute to psychological distress in frontline healthcare workers. In terms of clinical implications, our study highlights the urgency to create brief psychological interventions with promising efficacy that can be used in time-poor frontline settings to deal with the psychological distress of working in a frontline job [56,57].

There are a few limitations of the present study. First, given the cross-sectional nature of the study, no claims about causality could be made. To this end, further untangling the relationship of COVID-19 fear and COVID-19 stress (as independent variables) to anxiety and depression (as dependent variables) may prove complex. Future studies with longitudinal data are needed to examine the bidirectional effects that COVID-19 fear and COVID-19 stress may have on anxiety and depression, and vice-versa. Second, the present sample was limited to frontline health workers from three hospitals in the state of Selangor, which is in the main urbanized area in Malaysia and raises the issue of generalizability. Third, the ages in the present sample ranged from 23 to 57 years and were considered small to permit subgroup analyses. Future studies can focus on specific age groups and examine moderating effects that could identify those who are more likely to progress through the stages of psychological distress. Last but not least, given the extreme time pressures that frontline healthcare workers were under, we did not include other psychological distress determinants, such as burnout and trauma [58], in the present study to minimize test-taking fatigue. Taken together, future research studies that recruit longitudinal data from wider sociodemographic populations and that include other psychopathological variables in an attempt to predict psychological distress are strongly recommended.

## 5. Conclusions

Despite these limitations, the present study is the first to examine key constructs of fear and COVID-19 stress in relation to psychological distress in frontline healthcare workers who stand the highest risk for vicarious trauma from COVID-19 management. The present study is crucial in demonstrating that COVID-19 stress represents the key consistent factor that contributes significantly to anxiety and depression. The present findings suggest that future mental health research should focus on creating high-quality interventions with a clinical trial design to deal with the overwhelming burden of stress that comes unabated in successive waves of COVID-19 and with future pandemics. This line of research can then advise policymakers in creating more holistic national mental health policies that address frontline workers’ distress from a primary, rather than a tertiary, prevention point of view.

## Figures and Tables

**Table 1:** Descriptive and Bivariate Statistics.

Variables	1	2	3	4	5	6	7	8	9	10
1 Age	−									
2 Gender	−0.16 **	−								
3 Employment Status	0.29 **	0.08	−							
4 Shift Work	−0.27 **	0.19 **	−0.01	−						
5 Monthly household income ^log^	0.44 **	−0.18 **	0.08	−0.34 **	−					
6 Years of Employment ^log^	0.77 **	−0.10	0.40 **	−0.28 **	0.43 **	−				
7 COVID-19 Fear	−0.06	0.11	0.13 *	0.15 *	−0.20 **	−0.01	−			
8 COVID-19 Stress	−0.03	−0.07	−0.05	−0.01	0.04	0.01	0.51 **	−		
9 Anxiety	−0.08	−0.12 *	−0.10	−0.16 **	0.11	−0.07	0.30 **	0.58 **	−	
10 Depression	−0.21 **	−0.09	−0.12 *	−0.09	0.02	−0.20 **	0.30 **	0.53 **	0.81 **	−
*M*	33.02	0.80	0.84	0.52	3.73	0.76	17.44	8.78	3.74	6.66
*SD*	6.79	0.40	0.37	0.50	0.30	0.44	5.54	4.81	4.70	5.44
Skewness	1.36	−1.54	−1.89	−0.07	−0.06	−0.99	0.58	0.11	1.47	0.99
Kurtosis	1.92	0.38	1.59	−2.01	2.87	1.01	−0.02	−0.66	1.56	0.58

*Note.* Gender (1 = Female, 0 = Male), Employment Status (1 = Permanent Staff, 0 = Contract Staff), Shift Work (1 = Yes, 0 = No). ^log^ = log10-transformed data. * *p* < 0.05. ** *p* < 0.01.

**Table 2:** Summary of hierarchical multiple regressions predicting anxiety from sociodemographic and occupational variables.

**Variables**	β	t	$R^{2}$	$\Delta R^{2}$
Step 1			0.06	0.06
Age	−0.17 *	−2.50		
Gender	−0.10	−1.68		
Employment Status	−0.04	−0.67		
Shift Work	−0.14 *	−2.30		
Monthly Household Income ^log^	0.12	1.79		
Step 2			0.38	0.32
Age	−0.14 *	−2.53		
Gender	−0.07	−1.39		
Employment Status	−0.04	−0.82		
Shift Work	−0.15 *	−3.01		
Monthly Household Income ^log^	0.11	2.93		
COVID-19 Fear	0.10	1.63		
COVID-19 Stress	0.52 *	9.00		

*Note*. Gender (1 = Female, 0 = Male), Employment Status (1 = Permanent Staff, 0 = Contract Staff), Shift Work (1 = Yes, 0 = No). ^log^ = log10-transformed data. * *p* < 0.05.

**Table 3:** Summary of hierarchical multiple regressions predicting depression from sociodemographic and occupational variables.

**Variables**	β	t	$R^{2}$	$\Delta R^{2}$
Step 1			0.08	0.08
Age	−0.29 *	−4.24		
Gender	−0.10	−1.72		
Employment Status	−0.03	−0.44		
Shift Work	−0.11 *	−1.76		
Monthly Household Income ^log^	0.09	1.39		
Step 2			0.35	0.27
Age	−0.26 *	−4.53		
Gender	−0.07	−1.44		
Employment Status	0.03	−0.53		
Shift Work	−0.12 *	−2.28		
Monthly Household Income ^log^	0.08	1.44		
COVID-19 Fear	0.09	1.52		
COVID-19 Stress	0.46 *	7.89		

*Note*. Gender (1 = Female, 0 = Male), Employment Status (1 = Permanent Staff, 0 = Contract Staff), Shift Work (1 = Yes, 0 = No). ^log^ = log10-transformed data. * *p* < 0.05.

## Data Availability

The data that support the findings of this study are available on request from the corresponding authors. The data are not publicly available dues to privacy and/or ethical restrictions.
